# Distinct attention network topology and dynamics and their relations with pubertal hormones in preadolescent boys and girls with binge eating

**DOI:** 10.1038/s41398-025-03756-y

**Published:** 2025-11-22

**Authors:** Elizabeth Martin, Kurt P. Schulz, Tom Hildebrandt, Robyn Sysko, Laura A. Berner, Xiaobo Li

**Affiliations:** 1 Department of Biomedical Engineering, New Jersey Institute of Technology, 07102 Newark, NJ USA; 2 Department of Psychiatry, Icahn School of Medicine at Mount Sinai, 10029 New York, NY USA

**Keywords:** Neuroscience, Psychiatric disorders

## Abstract

The increased prevalence of binge eating during puberty is predominantly in girls, coinciding with a surge in pubertal hormones. This suggests that hormone-activated alterations in widespread brain networks, such as attention network, can contribute to the pathophysiology of the disorder in girls, while distinct mechanisms may exist in boys. This study proposed to examine the topological properties and their temporal dynamics of the sustained attention network in preadolescent children with binge eating symptoms (BE) and matched controls and to test the relation of these properties to circulating levels of pubertal hormones. Data from 77 children with BE and 104 group-matched controls were analyzed. In a static network comprising the entire task duration, the nodal topological properties, i.e., nodal efficiency, betweenness-centrality and degree, of the caudate nucleus, hippocampus and inferior parietal gyrus (IPG) significantly differentiated children with and without BE; and that of left caudate were significantly associated with pubertal hormone levels in girls with and without BE, but not in boys. During different substages of sustained attention processing, Variability of the topological properties in key network nodes, such as bilateral IPG, bilateral precentral gyrus and left hippocampus, demonstrated significant between-group differences and/or unique group-by-sex interactions. These results suggest that the association between pubertal hormones and network topological organization may contribute towards the specific rise of BE in girls, while neural mechanisms of BE in boys may alternatively link to suboptimal functional dynamics associated with precentral gyrus, during their interactions with other cortical and subcortical regions when sustained attention is performed.

## Introduction

Binge eating disorder (BED) is the most common eating disorder, affecting 1.1% of children and between 1 and 5% of adolescents [1, 2]. The risk for problematic binge eating (BE) increases from late childhood to early adolescence [1, 2], coincident with the pubertal surge of gonadal hormones [3] and may serve as a vulnerability for eating disorders in later life [4, 5]. However, the elevated risk is not spread uniformly, with a significantly greater increase in prevalence of BE during puberty in girls than boys [6]. This suggests that different neural developmental effects and pubertal hormones, as well as their distinct interactions, may be implicated in the pathophysiology of BE in boys and girls.

Widespread neural alterations have been identified in individuals with BE [7]. These include distinct patterns of dysconnectivity among distant brain regions or systems-level alterations of the functional brain networks subserving cognitive processes and/or at resting-state [8–11]. Several cognitive processes believed to be involved in BE, including attention, are supported by these widespread brain pathways and rely heavily on the maintenance of arousal [12–14]. Generalized brain arousal, which increases the excitation and activation of attention and behavior, may be implicated in these functional alterations [15].

The link between the pubertal hormone surge and generalized arousal has been established previously [16, 17]. These hormones have powerful direct and indirect influences on the noradrenergic pontine nucleus locus coeruleus that lies at the intersection of arousal and attention networks in the brain [18]. Estrogen binding to nuclear receptors in locus coeruleus enhances norepinephrine synthesis and reduces norepinephrine catabolism [19], thereby increasing synaptic levels of the neurotransmitter in coerulear terminal [20]. This in turn, drives neuronal activity in prefrontal regions that support attention [21] and basal forebrain regions that promote wakefulness [22].

This may contribute towards the increased vulnerability to BE during adolescence, particularly in girls, as gonadal hormones in particular estradiol, may drive the generalized arousal of the brain [23] and therefore, may serve as a vulnerability for BE in adolescence, especially in girls [24, 25]. Pubertal levels of the androgen testosterone and the estrogen estradiol have been divergently associated with ventral striatal activation and connectivity for reward responsivity and decision-making in young adolescent girls but not boys [26, 27]. While the aforementioned systems-wide alterations in brain function may point towards the aforementioned effects of generalized arousal, to date the relationship between pubertal hormones, and function in brain networks underpinned by generalized arousal underlying BE have not been investigated in children.

The current study proposed to assess firstly the systems-level topological properties and their temporal dynamics of the functional brain network subserving sustained attention processing in children with and without BE, and secondly the association of these functional brain properties to pubertal hormones. Graph theoretical techniques [28, 29] and sliding-window-based techniques [30, 31] were utilized to evaluate the topological properties of the functional brain network during a block-based attention task, as well as their temporal dynamics in the substages of attention initiation, stable attention, post-attention, and resting periods. Graph theoretical techniques allow the quantification of systems-level topological organization of the sustained attention network, by modelling the efficiency and connectedness of functional communications among all possible pairwise connections between remote brain regions in the network (network nodes) [28]. By analyzing the topological properties of a brain network highly dependent on generalized arousal, we aimed to link any observed alterations with the arousal-modulating pubertal hormone levels.

Our hypotheses are twofold: first, based on evidence of altered attentional processes [12, 14, 32] and of altered functional network properties in BE [11, 33], we hypothesized that relative to the matched control children, preadolescent children with symptoms of BE would show significant altered topological properties and their dynamics of the sustained attention network. Second, based on the association between pubertal hormones, generalized arousal and disordered eating risk, we further hypothesized that the topological property alterations in children with BE would be associated with levels of pubertal hormones, particularly in girls but not in boys.

## Methods

### Participants

Neuroimaging and clinical data from 77 children with BE and 104 matched controls were included in this study. These data were obtained from the Adolescent Brain Cognitive Development (ABCD) Study baseline pool (Release 4) and downloaded from the National Institute of Mental Health Data Archive. The ABCD Study aimed to recruit a sample that reflects the sociodemographic variation of the US population including race and ethnicity [34]. The baseline pool included 11,875 children aged 9 and 10 years, from 21 sites across the United States.

The original exclusion criteria of the ABCD Study baseline enrollment were: a current diagnosis of schizophrenia, autism spectrum disorder (moderate, severe), mental retardation/intellectual disability, or alcohol/substance use disorder [34]. To further remove potential confound in our findings, subjects with a history of traumatic brain injury or bipolar disorder were also excluded. Subjects with incomplete structural MRI, fMRI and/or task performance data, or low-quality imaging data (using the Human Connectome Project imaging data quality check criteria [35]), or missing/low quality pubertal hormone data [36] in both measurement repetitions were further excluded.

**Fig. 1:**
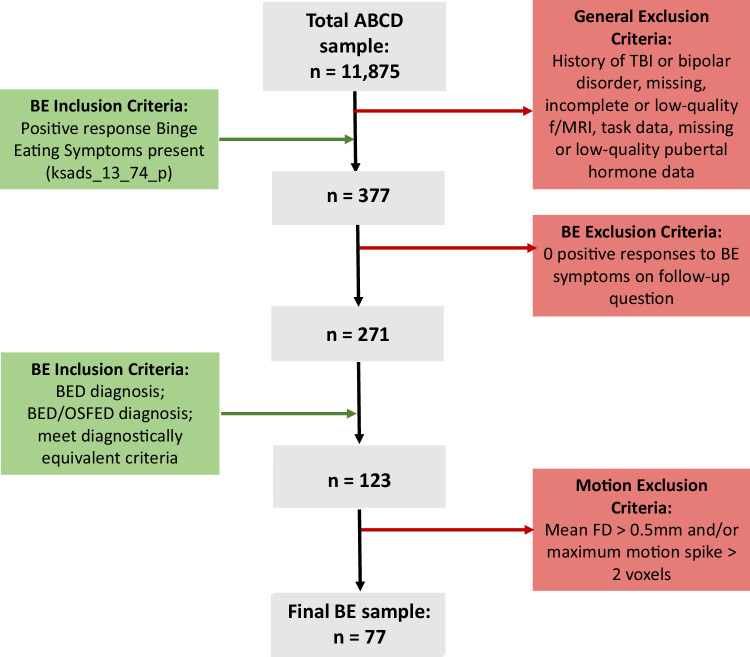
Binge eating participant inclusion/exclusion flowchart. Inclusion criteria are shown on the left (green), exclusion criteria are shown on the right (red), participants included at each stage shown in the middle (grey). BE, binge eating; BED, binge eating disorder; FD, framewise displacement; fMRI, functional magnetic resonance imaging; OSFED, otherwise specified feeding or eating disorder; TBI, traumatic brain injury.

A sample of children with BE was defined using multiple criteria, due to the complexity of diagnosis in pediatric BED. First, binge eating-related symptoms were assessed using the parent/guardian responses to the Kiddie Schedule for Affective Disorders and Schizophrenia (K-SADS) based on DSM-5 criteria [37]. An initial pool of 377 children with present binge eating were identified, based on parent reports indicating presence of binge eating (ABCD K-SADS item: Symptom - Binge Eating Present). From this initial pool, 106 subjects were excluded due to parents reporting ‘no’ to all 7 items in the binge eating supplementary scale used to characterize individual binge eating behaviors (e.g. “My child eats a lot even though he or she is not hungry”, “My child feels disgusted or guilty after binge eating”). In the remaining 271 subjects with present binge eating diagnosis/behaviors, we defined a group of 123 as having BE using the following inclusion criteria: i) having a BED diagnosis (*n* = 37); ii) having a diagnosis of BED/Other Specified Feeding or Eating Disorders/not meeting full diagnostic criteria (*n* = 28); or iii) no diagnosis but reported at least one binge eating episode per week for at least 3 months (diagnostically equivalent to full-threshold BED criteria) plus parent report of child displaying at least one binge eating behavior present on the KSADS (*n* = 58). Among the 123 subjects with BE, 46 were excluded from group-level analyses due to excessive head movements in the fMRI data (see below in the *Imaging Data Acquisition and Preprocessing* Section). The flow of BE group identification process is graphically depicted in Fig. 1.

A pool (*n* = 2340) of control participants was identified following the ABCD Study and the current study-specific exclusion criteria, without diagnosis of any eating disorder, and no participant or family history of psychiatric disorders. A study-specific control group was then defined from this pool, consisting of 104 participants, who were pseudo-randomly selected from participants with complete and high quality imaging data with head motion passing the motion-based criteria described in the Section of *Imaging Data Acquisition and Preprocessing*, and matched on age, height, IQ (age-corrected picture vocabulary), sex, handedness and combined parental income (to reflect socioeconomic status) to the group with BE.

As our sample size was determined by available data, we ran a power analysis to ensure the final sample size was sufficient, particularly to detect interaction effects. The power for a 2 × 2 interaction assuming rX = 0.45 and rX2 = 0.2 (one medium effect and one small effect on Y) and a correlation of r =0.4 between them (moderate correlation of predictors) and examining effects of the interaction ranging from 0.10 to 0.45 (small to medium effect), we estimated power with total sample size ranging from 100 to 700 using 10,000 simulations in InteractPowerR v0.2.2. We achieved at least 80% power with interaction effects at 0.25 with sample size of 100 [38]. For the main group effect we estimated power with total sample size ranging from 20 to 200 and achieved at least 80% power with sample size of 100. Figures S1 and S2 summarize our simulations.

### Ethics approval and consent to participate

The ABCD Study was approved by the institutional review board (IRB) of the University of California, San Diego and of each data collection site. Informed consent and informed assent were obtained from parents and participants, respectively. The current study is a secondary analysis of de-identified data and therefore IRB approval was waived.

### Pubertal hormones

Testosterone, dehydroepiandrosterone (DHEA) and estradiol (in girls only) levels were obtained at ABCD study sites by salivary measurement. Details of the measurement procedures are described elsewhere [39]. Briefly, saliva was collected via passive drool method following 30 min of no eating, drinking or chewing of gum. Saliva samples were frozen on site and analyzed in duplicate by Salimetrics (Carlsbad, CA). Raw hormone assay values were standardized on age, and Pubertal Development Scale (PDS) subscale score, by sex using Bayesian mean estimation with priors derived from population age and race ethnicity norms. The remaining DHEA, testosterone, and estradiol values were standardized within the Bayesian model by averaging across age weighted for PDS subscale and derived by controlling for a number of factors that can impact hormone level: i) time of day of saliva collection; ii) color of the sample; iii) duration of sample collection; iv) caffeine and v) vigorous physical activity. Pubertal stage was estimated using the PDS completed by the parent. Pubertal stage in girls was calculated by summing responses for body hair and breast growth, combined with information about menarche. Pubertal stage in boys was calculated by summing responses for body hair, facial hair and voice changes [36, 40]. Final hormone values used in analysis were single values derived from averaging Bayesian estimates of each repetition.

### Imaging data acquisition and preprocessing

We utilized fMRI data collected during the 0-back blocks of the emotional n-back task [41] (see supplementary material for full task description, and Table 1 for behavioral performance). Unlike the 2-back trials of the task which require working memory, the 0-back trials instead require only sustained attention without a working memory loading, and alone are sometimes referred to as the attentional 0-back task [41–43]. Contrasting 0-back trials with fixation trials shows significant activation in bilateral cortical regions involved in the dorsal and ventral attention networks (DVAN), including several regions of the frontal cortex, the inferior parietal gyrus (IPG), and subcortical regions, including the dorsal and ventral striatum and the thalamus [44].

**Table 1:** Participant characteristics and behavioral performance.

	BE *N* = 77	CON *N* = 104	T	P
	M(SD)	M(SD)		
***Age***	9.95 (0.62)	9.97 (0.60)	0.25	0.80
***IQ***	99.1 (11.8)	101.7 (16.1)	0.86	0.38
***BMI percentile***	0.89 (0.90)	0.67 (0.28)	6.0	<0.001
***0-back n trials correct***	64.5 (13.3)	66.8 (10.4)	-1.3	0.19
***0-back RT***	917.2 (140.6)	912.7 (122.0)	0.23	0.82
***0-back SDRT***	295.2 (59.1)	288.6 (55.1)	0.77	0.44
	**n**	**n**	**X**^**2**^	**P**
***Sex***			0.64	0.42
Girls	44	52		
Boys	33	52		
***Scanner Type***			1.4	0.52
Siemens	50	34		
Phillips	10	68		
GE	17	31		
***Race***			13.6	0.057
American Indian/ Alaska Native	0	3		
Asian	0	1		
Black/ African American	15	12		
Mixed/ Other	23	16		
Native Hawaiian/ Pacific Islander	0	1		
White	35	65		
No Response	3	4		
***Ethnicity***			0.22	0.90
Hispanic	27	34		
Non-Hispanic	49	68		
No Response	1	2		
***Pubertal Stage***			10.2	0.07
Pre	23	46		
Early	15	23		
Mid	31	24		
Late	0	2		
Post	1	0		
***Handedness***			4.9	0.09
Left	8	10		
Right	53	98		
Mixed	16	15		
***Parental Income***			8.6	0.47

*BE* participants with binge eating, *CON* control, *IQ* intelligence quotient (age-corrected picture vocabulary), *BMI* body mass index, *RT* reaction time, *SDRT* standard deviation of the reaction time.

Detailed data acquisition protocols from the ABCD Study have been published elsewhere [41]. Briefly, two runs of the task-based scans lasting approximately 5 min were acquired using whole-brain multiband echo planar imaging depicting the blood oxygenation level-dependent (BOLD) signals (TR = 800 ms, TE = 30 ms, flip angle = 30°, slices = 60, resolution = 2.4 × 2.4 × 2.4, multiband acceleration factor = 6).

Raw fMRI data was downloaded and preprocessed using the FEAT toolbox (FMRIB Software Library, FSL, version 5.0). First, the initial volumes were removed from the time series, depending on the manufacturer (Philips and Siemens original n volumes = 370 and n removed = 8; GE DV25 series original n volumes = 367 and n removed = 5, GE DV26 s series original n volumes = 378 and n removed = 16). Next, slice time correction, and motion correction using MCFLIRT were performed on the time series, and a high-pass temporal filter with a 100-second cut-off (0.01 Hz) was applied to remove slow frequency drifts. Spatial smoothing using a 5 mm gaussian kernel was then applied. Finally, functional images were spatially normalized to a standard MNI space pediatric image [45], with a resolution of 1 ×1 x 1 mm. Functional connectivity and network analyses are particularly sensitive to excessive motion [46, 47], and therefore stringent motion-based exclusion criteria were applied in this study. Subjects were excluded if framewise displacement [46] in both task runs exceeded 0.5 mm and/or if maximum motion exceeded 2 voxels in any direction (See Fig. 1 for subjects excluded).

### Node selection and BOLD timeseries extraction

**Fig. 2:**
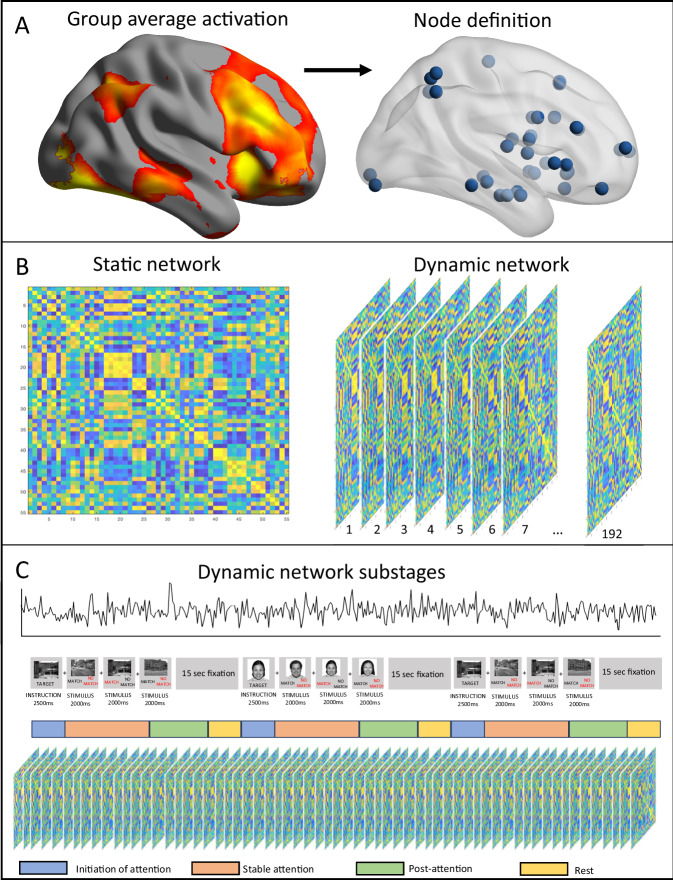
Analysis pipeline. **A**: Nodes are defined first by calculating a group-average, then 4 mm spherical nodes are defined around activation peaks. **B** Correlation matrices (55 ×55) are calculated to reflect connections (Pearson correlation) between each node. The matrix is binarized to create a network. This is either done once (static network) or 192 times (sliding window dynamic network). For each network created, nodal global efficiency, degree and betweenness centrality are calculated. **C** Breakdown of concatenated sustained attention trials into four dynamic stages: initiation, stable attention, post-attention and rest.

A combined activation map of the two groups was first generated for the contrast of interest (0-back versus fixation) (see Fig. 2A). This reference activation map was parcellated into 246 distinct regions using the Brainnettome Atlas [48]. Network nodes were defined as 4-mm spheres around peak activation in clusters thresholded at *T* ≥ 2.3 in each region with at least 800 voxels of contiguous activation, and in the bilateral pulvinar nucleus. This resulted in 55 network nodes located in bilateral cortical and subcortical areas from the reference activation map. For each subject, the BOLD signals of each node (averaged over voxels in each node) were extracted from a sequence of 208 brain volumes consecutively formed along each 0-back block and the rest block immediately following the 0-back block. To further remove head motion-related artifact in the signals, a six-level wavelet noise filtering process was applied to the resulting timeseries of each node, using the inverse Maximal Overlap Discrete Wavelet Transform with the Symlet wavelet family, in the GAT-FD toolbox [49, 50].

### Static functional network construction

A 55 ×55 functional connectivity matrix was then calculated for each subject using Pearson’s correlation of the BOLD signal timeseries in each pair of the network nodes. The connectivity matrix was binarized by using network cost as thresholds, defined as the fraction of existing edges relative to all possible edges in a network [51]. The appropriate cost range for thresholding the network was then determined to be between 0.14 and 0.45 and the topological properties of the network were calculated within this range at an increment of 0.1, at each increment retaining positive edges that exceed the threshold only. Details and justification of the determination of the appropriate cost range can be found in Supplementary Materials. The network global and local efficiencies were calculated for the overall network. The nodal efficiency, nodal degree and betweenness-centrality (BC) were obtained for each network node. Mathematical definitions of these topological properties can be found in Supplementary Materials. Neurobiologically, the network global efficiency reflects the ability of the network to efficiently transfer information across all distributed network nodes (i.e. the network as a whole). Network local efficiency reflects the network’s efficiency/speed for information transfer between neighboring nodes. Nodal efficiency reflects the efficiency/speed with which any given node communicates with all other connected nodes, while nodal BC and nodal degree are measures of the extent of connectedness to other nodes in the network, or hubness of a given node.

### Network dynamics

Functional connectivity of brain regions can vary during processes of sensory and cognitive tasks or even in resting-state [30, 52]. An increasing number of studies suggest that temporal variance of functional brain networks contributes to cognitive performance, and alterations in this variance may play a significant role in neuropsychiatric conditions [31]. For example, while increased variance in networks has been associated with superior cognitive performance [53, 54] both increased and decreased network temporal variance is reported in schizophrenia, major depression and bipolar disorder [31]. To analyze the dynamics of the topological properties of the DVAN, we utilized the sliding window-based techniques from the GAT-FD toolbox [55]. A temporal window of 17 volumes was used with a sliding window step size of one volume. This resulted in a total of 192 temporal windows per task run per subject, and a network was constructed for each window. The dynamic network threshold was correlation coefficients between 0.24 and 0.78, corresponding to the top 45-15% strongest functional connectivity. This threshold range should retain the connections meeting the small-world network assumptions [50].

The first seven networks in each run were discarded to account for the slow BOLD response and fast repetition time (800 ms). The remaining 185 networks were then classified as during the substages of stable attention (n networks = 60), post-attention (n networks = 48), rest (n networks = 32) and attention initiation (n networks = 45), based on the temporal locations of the brain networks along the task (see Fig. 2B and C). To assess the temporal dynamics and variability of the topological properties of the DVAN along the task duration, Variance across each substage was calculated for nodal efficiency, BC and degree were calculated for each network node for each of the four substages. Variance was defined as:

$${\rm{Variance}}=\frac{1}{N-1}\mathop{\sum }\limits_{i=1}^{N}{{|A}i-\mu |}^{2}$$

Where *A* is a vector made up of *N* observations (i.e. networks) and *μ* is the mean of *A*.

### Group-level statistical analysis

Participant characteristics including basic demographic information and pubertal stage were analyzed using t-test and chi-square tests for continuous and categorical data, respectively. Behavioral performance during the 0-back blocks of the task was analyzed using a t-test to assess group differences in accuracy, mean reaction time, and standard deviation of reaction time.

To analyze static and dynamic network properties, the following ANCOVA model was tested:

$${Node\; Property} \sim {group}+{BMI} \% +{SES}+{IQ}+{sex}+{group}\times {sex}$$

Homogeneity of variance was confirmed using Levene’s test (*p* > 0.05) and normality was confirmed by inspection of Q-Q plots. Results were considered in the two global network properties and the nodal properties in 15 key cortical and subcortical brain components, determined based on those regions defined as belonging to the DVAN [56, 57], including bilateral superior frontal gyri (SFG), middle frontal gyri (MFG), IPG, insula, inferior temporal gyri as well as the pulvinar nuclei of thalamus and dorsal striatum. Bilateral hippocampi were also included in analyses. Although hippocampus is not a core structure of the DVAN, there is solid evidence suggesting that it interacts with these networks through its role in memory retrieval, where it may be activated when attention needs to access stored information from memory, especially through connections with the dorsal attention network which manages top-down attentional control [58, 59]. Bonferroni correction was applied over results from these nodes.

A partial least squares (PLS) regression was used (using MATLAB function plsregress) to assess the relationship between pubertal hormones (testosterone, DHEA and estradiol in girls, testosterone and DHEA in boys) and the nodal network properties showing significant (uncorrected) group differences. For any given subject, covariance between network properties across the involved network nodes, as well as between circulating levels of pubertal hormones is expected. PLS regression allows assessment of the relationship between pubertal hormones and network properties by creating uncorrelated components, thus solving the issue of collinearity in these measurements [60]. The PLS latent brain component explaining a significant percentage of variance was identified separately for boys and girls with and without BE, as boys do not have data on estrogen levels. The loadings for the significant brain component were compared for the different groups. Significance of PLS components was tested using permutation with the number of iterations justified based on the sample size (n permutations = 500). Variable Importance in Projection (VIP) scores were calculated for each brain region. Brain components with a VIP scores > 1 are considered important in the association between brain and hormonal components [61].

## Results

### Demographic measures

Participants with BE were in a significantly higher BMI percentile (95% CI: 0.14 – 0.29, *p* <0.001) than control participants. There were no significant group differences in sociodemographic measures, pubertal stage or scanner type. Statistical comparisons of participant characteristics are shown in Table 1.

### Behavioral performance

There were no significant differences between BE and control children in 0-back accuracy (95% CI: -5.91 – 1.19; *p* = 0.19), reaction time (95% CI: -34.86 – 43.96; *p* = 0.82) or standard deviation of reaction time (95% CI: -10.49 – 23.89; *p* = 0.44). Group statistics for behavioral performance can be found in Table 1.

*Pubertal Hormones* There were no significant differences between BE and control children in pubertal hormone levels (all *p* > 0.05).

In participants with BE, the mean testosterone level for boys was 32.6 pg/mL ( ± 16.6 pg/mL), and for girls, it was 45.6 pg/mL ( ± 23 pg/mL). For DHEA, the average level in boys was 62 pg/mL ( ± 36 pg/mL), while for girls, it was 102 pg/mL ( ± 60 pg/mL). In girls with BE, the mean estradiol level was 1.14 pg/mL ( ± 0.5 pg/mL).

In control participants, the mean testosterone level for boys was 34.4 pg/mL ( ± 16.8 pg/mL) and for girls, it was it was 37.5 pg/mL ( ± 14 pg/mL). For DHEA, the average level in boys was 69.2 pg/mL ( ± 51.9 pg/mL) while for girls, it was 76 pg/mL ( ± 42 pg/mL). In control girls, the mean estradiol level was 1.03 pg/mL ( ± 0.5 pg/mL).

### Static network topological properties

There were no significant differences between BE and control children in network global efficiency or network local efficiency (*p* > 0.05). Group means of all the significant nodal properties prior to Bonferroni correction are included in Table 2. Children with BE showed significantly higher nodal efficiency in left hippocampus (95% CI: 6.99 – 12.90; *p*_*uncorrected*_ = 0.002, *p*_*BON*_ = 0.03, η²p = 0.0276) and left caudate (95% CI: 12.84 – 23.67; *p*_*uncorrected*_ <0.001, *p*_*BON*_ = <0.001, η²p = 0.0494); significant higher nodal degree in left hippocampus (95% CI: 7.0 – 12.99; *p*_*uncorrected*_ = 0.002, *p*_*BON*_ = 0.03, η²p = 0.0277) and left caudate (95% CI: 8.67 – 15.99; *p*_*uncorrected*_ <0.001, *p*_*BON*_ = 0.01, η²p = 0.0338); and significant higher nodal BC in left IPG (95% CI: 7.69 – 14.169; *p*_*uncorrected*_ = 0.001, *p*_*BON*_ = 0.015, η²p = 0.031). The BMI percentile covariate significantly contributed to the model for left caudate efficiency (*p* = 0.028), left hippocampus degree (*p* = 0.045) and left caudate degree (*p* = 0.046). However, these effects did not survive Bonferroni correction. Significant group-by-sex interactions of the static topological properties were not observed in any nodes within the DVAN (all *p* > 0.05). Group means for all significant nodal properties prior to Bonferroni correction are included in Table S1.

**Table 2:** Group Differences in Static Network Properties.

	Region	BE vs CON	BE M(SD)	CON M(SD)	p_uncorrected_	p_BON_	partial eta^2^
***Efficiency***							
	L Hippocampus	↑	0.48 (0.11)	0.43 (0.12)	0.002	0.03	0.0276
	L Caudate	↑	0.57 (0.08)	0.55 (0.10)	<0.001	<0.001	0.0494
***Degree***							
	L Hippocampus	↑	10.8 (5.5)	8.6 (4.7)	0.002	0.03	0.0277
	L Caudate	↑	16.4 (4.9)	15.1 (5.9)	<0.001	0.01	0.0338
***BC***							
	L Inferior Parietal Gyrus	↑	64.0 (58.6)	41.8 (38.7)	0.001	0.015	0.031

*L* left, *R* right, *BE* participants with binge eating, *CON* control, P_BON_ p-values bonferroni corrected for network, Symptoms, *BC* betweenness-centrality. significant results following bonferroni correction are shown in bold.

### Dynamics of the nodal topological properties across substages of attention processing

#### Attention initiation substage

Following Bonferroni correction, children with BE showed significantly reduced Variance of nodal degree during the initiation of attention in right IPG (95% CI 6.47 – 11.93*, p*_*uncorrected*_ = 0.003, *p*_*BON*_ = 0.045, η²p = 0.026) compared to control children, and there was a significant group-by-sex interaction in Variance of nodal efficiency in right precentral gyrus (PrG). The BMI percentile covariate did not significantly contribute to the model for these measures during attention initiation (all *p* > 0.05). Post-hoc tests revealed reduced Variance of right PrG nodal efficiency in boys with BE compared to control boys (95% CI -0.004 – -0.0009, p = 0.002, Cohen’s d = -0.62). There was no difference in the variability of PrG nodal efficiency between girls with and without BE (*p* > 0.05).

#### Stable attention substage

Children with BE showed significantly increased Variance of nodal BC during stable attention in right middle frontal gyrus (95% CI 6.85 – 12.63*; p*_*uncorrected*_ = 0.003, *p*_*BON*_ = 0.045, η²p = 0.027) compared to control children, and there was a significant group-by-sex interaction in the Variance of the right PrG following Bonferroni correction. The BMI percentile covariate did not significantly contribute to the model for these measures during stable attention (*p* > 0.05). Post-hoc tests revealed reduced Variance of right PrG degree in boys with BE compared to control boys (95% CI -11.6 – -0.07, p = 0.047, Cohen’s d = -0.40). There was no difference in PrG efficiency between BE and control girls (*p* > 0.05).

#### Post-attention Substage

Analyses revealed no significant differences between BE and control children or group-by-sex interactions in the Variance of the nodal topological properties in any nodes of the DVAN during the post-attention substage (all *p* > 0.05).

#### Rest substage

Children with BE showed significantly increased Variance of left IPG BC during rest (95% CI 6.66 – 12.28*; p*_*uncorrected*_ = 0.003, *p*_*BON*_ = 0.045, η²p = 0.026) compared to control children, and there were significant group-by-sex interactions in the Variance of right PrG nodal efficiency (*p*_*BON*_ = 0.015) and left hippocampus BC (*p*_*BON*_ = 0.03), all survived Bonferroni correction. The BMI percentile covariate did not significantly contribute to the model for these measures during stable attention (*p* > 0.05). Post-hoc T-tests showed increased Variance of nodal efficiency in PrG in girls with BE compared to control girls (95% CI 0.0003 – 0.003, p = 0.015, Cohen’s d = 0.50), while in contrast, Variance of right PrG nodal efficiency was lower in boys with BE than control boys (95% CI -0.004 – -0.0004, p = 0.017, Cohen’s d = -0.47). Post-hoc tests also revealed increased Variance in hippocampal BC in girls with BE compared to control girls (95% CI 444.9 – 2172.2, p = 0.003, Cohen’s d = 0.62). There was no difference in hippocampal BC between boys with and without BE (*p* > 0.05).

Results of analyses in network dynamics during all substages of attention processing are reported in full in supplementary Tables S2 and S3, including all significant nodal properties prior to and post-Bonferroni correction.

### Relationship with hormones

**Fig. 3:**
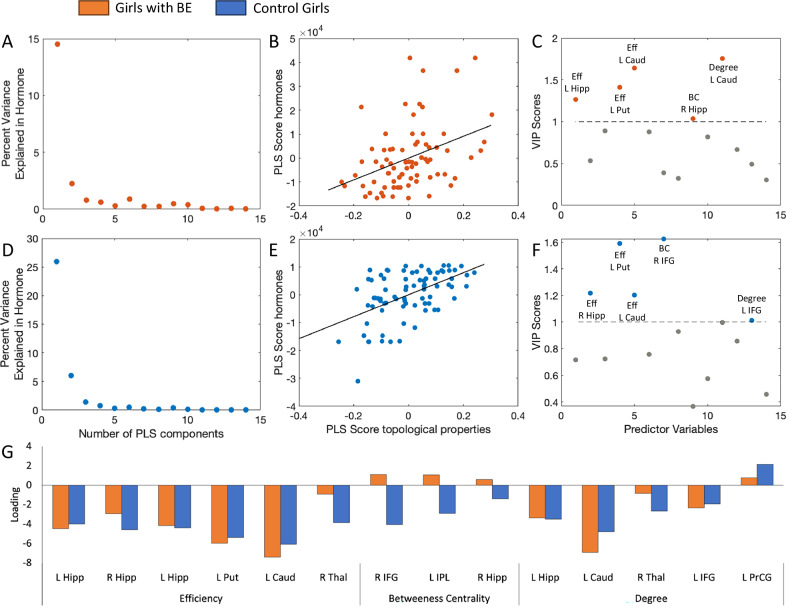
PLS Regression Analysis of Gonadal Hormones and Attention Network Properties. **A**-**C** show the significant first PLS component and the significant correlation between hormone PLS scores and network properties PLS scores and VIP scores for the brain predictor variables for girls with BE (orange) and **D-F** show the equivalent for control girls (blue). **G** shows the network property loadings onto the significant PLS component.

The first brain PLS component explained a significant amount of variance in the hormone measures in both girls with BE (p_perm500_ = 0.021) and control girls **(**p_perm500_ < 0.001). Weightings of factor loadings separately for girls with and without BE can be found in Fig. 3. For both groups of girls, the strongest factor loading was a negative loading for nodal efficiency in left caudate nucleus and left putamen and a negative loading for nodal degree in left caudate nucleus. The first PLS component did not explain a significant amount of variance in the hormone measure for either boys with BE or control boys (p_perm500_ = 0.69 and p_perm500_ = 0.052 respectively). In BE girls, efficiency in the left hippocampus, the left putamen and left caudate, BC in the right hippocampus and degree in the left caudate had VIP scores > 1. In control girls, efficiency in the right hippocampus, the left putamen and left caudate, BC in the right IFG and degree in the left IFG had VIP scores > 1. VIP scores are presented in Fig. 3.

## Discussion

The results of this study demonstrated that during sustained attention processing, the topological properties and their temporal dynamics associated with several key nodes of the DVAN distinguished children with BE from control children. Specifically, children with BE showed significantly increased nodal efficiency and degree of the functional subnetworks associated with left caudate and left hippocampus and increased BC of the subnetwork associated with left IPG during the entire task period; as well as significantly altered dynamic variability of these nodal topological properties of the subnetworks associated with bilateral IPG and right MFG. Meanwhile, the nodal efficiency and degree of left caudate, in which between-group differences were identified, contributed to an association between circulating pubertal hormones and functional brain integrity in girls only, suggesting a female-specific pubertal-hormone dependent mechanism via altered efficiency and connectedness of left caudate during its functional communications with other brain regions for sustained attention processing. Alternatively, boys with BE were distinguished from control boys through reduced dynamic variability of nodal efficiency and connectedness of the subnetwork associated with of bilateral PrG. These sex-specific findings suggest differentiating mechanisms underlying binge eating in preadolescent boys and girls.

Participants with BE were distinct from control participants in several measures, notably through consistent differences associated with IPG, in both the topological properties and their functional dynamics along different substages of the attention task. During the entire task, children with BE showed increased overall speed for functional information transferring and hyper-communications among brain regions in the subnetwork associated with left IPG. Furthermore, the dynamic variability of speed for information transferring and connectedness of the subnetwork associated with IPG in children with BE were found to be overly decreased during the attention initiation substage, while abnormally increased during resting-state. IPG is a major hub of the fronto-parietal system and plays a critical role in a range of cognitive functions, including attention [62]. Alterations in regional activation and seed-based connectivity in IPG have previously been implicated in binge eating, with reports of both increased and decreased functional connectivity in this region in participants with BED [10, 63], possibly contributing to the posited role of attentional processes in the disorder [13, 14, 32]. Our observation of differences in the functional organization of this junction of auditory and visual processing streams may indicate widespread impacts on functioning in children with BE, as would be expected if generalized arousal was impacted. Interestingly, the overall betweenness-centrality of left IPG loaded in opposite directions on the pubertal hormone component for girls with and without BE, with the former group showing a small, positive loading versus a stronger, negative loading in the latter group. This suggests that the relationship between circulating pubertal hormones and functional brain properties associated with IPG is distinct in girls with and without BE, and may contribute towards BE in girls.

Our results in girls support the hypothesized association between pubertal hormone levels and the function of a central node in the ventral attention network that may convey vulnerability for BE in young adolescent girls specifically. The finding of this association in girls but not boys suggests that estradiol actions at estrogen receptors may contribute towards this relationship. Caudate nucleus plays a critical role in goal-directed behavior through the prioritization of actions based on outcome [64] and has been implicated in the control of food intake [65, 66], with regional caudate fMRI activation positively associated with binge eating severity [10, 67]. Caudate spiny projection neurons and interneurons all express membrane-bound estrogen receptors [68], which increases neuronal excitability beginning during puberty [69] and impedes the synaptic plasticity that supports learning [70]. Estradiol indirectly promotes basal and stimulated dopamine release and dopamine receptor expression in dorsal striatum [71], and is linked to increased nucleus accumbens activation for decision-making in adolescent girls [27] and reduced caudate responses to reward cues. The significant associations of pubertal hormone levels in girls to the nodal efficiency and connectedness of left caudate nucleus suggest that, in early pubertal developmental stages, pubertal hormone levels are associated with organization of BE-relevant neurocircuitry [24, 25]. Although we did not find group differences in levels of pubertal hormones, this absence of significant difference may be due to the early pubertal developmental stages of participants. As pubertal development continues, significant differences in circulating pubertal hormones may become evident between adolescents with and without binge eating [6] and further contribute towards functional network alterations. The general arousing effects of the increasing pubertal levels of estradiol may disrupt the prioritization of actions in the caudate nucleus [64], although due to the cross-sectional nature of the evidence presented here, longitudinal research is required to test any lasting organizational impacts into adulthood of estradiol levels in puberty. Additionally, future research should consider whether similar relationships between pubertal hormone and network organization exist in bulimia nervosa, for which there is also a reported sex difference in prevalence [72], to understand if the associations identified here reflect a neurobiological risk for the transdiagnostic behavior of binge eating.

Intriguingly, the temporal dynamic patterns of topological properties associated with bilateral PrG showed significant group-by-sex differences in multiple substages of sustained attention processing. Particularly, boys with BE showed significantly reduced dynamic variability of nodal efficiency and degree of right PrG in attention initiation and resting substages, and reduced dynamic variability of nodal degree of left PrG during the stable attention substage. Girls with BE showed differences in variance in right PrG efficiency in the rest substage only, interestingly in the opposite direction to boys. PrG activation has been consistently reported to be associated with food motivation in children and adolescents [73], and differences of functional activation in this region have previously been observed in adults with BED compared to control adults [63]. The sex-specific distinct patterns of altered temporal dynamics of PrG-associated subnetwork suggest sex-specific differences in children with BE in the propensity for the DVAN to maintain its performance during different attentional substages. Specifically, consistent differences in boys with BE compared to control boys point towards a mechanisms contributing to binge eating in boys that does not consistently exist in girls, distinct from the sex-specific hormone-driven mechanism discussed above. It should be noted that group-by-sex interactions of the static topological properties (overall averaged across the entire task duration) associated with PrG were not observed in the analyses. While to-date there is not consensus about the direct impacts of reduced network variability, our results suggest that advanced investigations into the temporal dynamics of functional brain networks subserving sensory/cognitive processes could provide us more refined evaluations of the neurophysiological mechanisms associated with BE; while on the other hand, the relationship between sex, BE, and PrG properties requires further investigation.

### Limitations

This study has limitations that need to be considered. First, as the study is cross-sectional, we cannot conclude any potential developmental effects of pubertal hormones on the DVAN organization. The majority of the sample used here were pre-, early- or mid-pubertal. It is likely that the influence of pubertal hormones will develop into adolescence, as pubertal development continues. Further studies should utilize the longitudinal nature of the ABCD study to assess how the relationships identified here cross-sectionally develop with age. Second, although sustained attention is dependent upon generalized arousal, we make conclusions here relating to generalized brain arousal using sustained attention as a proxy measure. While we believe that the relationship between systems-level brain alterations and pubertal hormones likely arises as a result of the known effect of pubertal hormones on generalized arousal, it is possible that these associations arise reflect more specific or localized effects. Finally, although we controlled for BMI percentile in our regression models, children with BE were in a significantly higher BMI percentile than control children, and this may have contributed towards group differences observed.

## Conclusions

We observed for the first time alterations in topological properties of the DVAN during sustained attention in children with BE, supporting the evidence for alterations in attentional processes in BED. We identified an association between circulating hormones and nodal topological properties of the attention network in girls but not boys, which may be reflective of the impact of estrogen on widespread circuitry underlying generalized brain arousal, which in turn may contribute towards the increased risk for binge eating in girls during puberty by impacting widespread brain pathways. Alternatively, boys with BE showed consistent alterations associated with the PrG, potentially reflecting a distinct mechanism. Future longitudinal work will further elucidate the sex-specific mechanisms underlying binge eating and the impacts of gonadal hormones on binge eating-related neurocircuitry across pubertal development.

## Supplementary information

Supplementary Materials

## Data Availability

All relevant data for this work are available from the corresponding authors upon reasonable request.

## References

[1] Marzilli E, Cerniglia L, Cimino S. A narrative review of binge eating disorder in adolescence: prevalence, impact, and psychological treatment strategies. Adolesc Health Med Ther. 2018;9:17–30. 10.2147/AHMT.S148050. 29379325 10.2147/AHMT.S148050 PMC5759856

[2] Murray SB, Ganson KT, Chu J, Jann K, Nagata JM. The prevalence of preadolescent eating disorders in the United States. Journal Adolesc Health. 2022;70:825–8. 10.1016/j.jadohealth.2021.11.031. 10.1016/j.jadohealth.2021.11.031 35078736

[3] Farello G, Altieri C, Cutini M, Pozzobon G, Verrotti A. Review of the literature on current changes in the timing of pubertal development and the incomplete forms of early puberty. Front Pediatr. 2019;7:147 10.3389/fped.2019.00147. 31139600 10.3389/fped.2019.00147 PMC6519308

[4] Brewerton TD, Rance SJ, Dansky BS, O’Neil PM, Kilpatrick DG. A comparison of women with child-adolescent versus adult onset binge eating: Results from the National Women’s Study. International J Eat Disord. 2014;47:836–43. 10.1002/eat.22309. 10.1002/eat.22309 24904009

[5] Tanofsky-Kraff M, Shomaker LB, Olsen C, Roza CA, Wolkoff LE, Columbo KM, et al. A prospective study of pediatric loss of control eating and psychological outcomes. J Abnorm Psychol. 2011;120:108–18. 10.1037/a0021406. 21114355 10.1037/a0021406 PMC3051193

[6] Mikhail ME, Anaya C, Culbert KM, Sisk CL, Johnson A, Klump KL. Gonadal Hormone influences on sex differences in binge eating across development. Curr Psychiatry Rep. 2021;23:74. 10.1007/s11920-021-01287-z. 34613500 10.1007/s11920-021-01287-z PMC8576863

[7] Leenaerts N, Jongen D, Ceccarini J, Van Oudenhove L, Vrieze E. The neurobiological reward system and binge eating: A critical systematic review of neuroimaging studies. International J Eat Disord. 2022;55:1421–58. 10.1002/eat.23776. 10.1002/eat.23776 35841198

[8] Murray SB, Alba C, Duval CJ, Nagata JM, Cabeen RP, Lee DJ, et al. Aberrant functional connectivity between reward and inhibitory control networks in pre-adolescent binge eating disorder. Psychol Med. 2023;53:3869–78. 10.1017/S0033291722000514. 35301976 10.1017/S0033291722000514

[9] Stopyra MA, Simon JJ, Skunde M, Walther S, Bendszus M, Herzog W, et al. Altered functional connectivity in binge eating disorder and bulimia nervosa: A resting-state fMRI study. Brain Behav. 2019;9:e01207. 10.1002/brb3.1207. 30644179 10.1002/brb3.1207 PMC6379643

[10] Haynos AF, Camchong J, Pearson CM, Lavender JM, Mueller BA, Peterson CB, et al. Resting state hypoconnectivity of reward networks in binge eating disorder. Cereb Cortex. 2021;31:2494–504. 10.1093/cercor/bhaa369. 33415334 10.1093/cercor/bhaa369 PMC8248831

[11] Martin E, Cao M, Schulz KP, Hildebrandt T, Sysko R, Berner LA, et al. Distinct topological properties of the reward anticipation network in preadolescent children with binge eating disorder symptoms. J Am Acad Child Adolesc Psychiatry. 2024;63:1158–68. 10.1016/j.jaac.2024.02.015. 38461893 10.1016/j.jaac.2024.02.015 PMC11380707

[12] Kaisari P, Dourish CT, Rotshtein P, Higgs S. Associations between core symptoms of attention deficit hyperactivity disorder and both binge and restrictive eating. Front Psychiatry. 2018;9:103 10.3389/fpsyt.2018.00103. 29651258 10.3389/fpsyt.2018.00103 PMC5884932

[13] Lyu Z, Zheng P, Jackson T. Attention disengagement difficulties among average weight women who binge eat. European Eat Disord Rev. 2016;24:286–93. 10.1002/erv.2438. 26856539 10.1002/erv.2438

[14] Halevy-Yosef R, Bachar E, Shalev L, Pollak Y, Enoch-Levy A, Gur E, et al. The complexity of the interaction between binge-eating and attention. PLoS ONE. 2019;14:e0215506. 10.1371/journal.pone.0215506. 31017971 10.1371/journal.pone.0215506 PMC6481844

[15] Pfaff D, Ribeiro A, Matthews J, Kow L-M. Concepts and mechanisms of generalized central nervous system arousal. Ann N. Y Acad Sci. 2008;1129:11–25. 10.1196/annals.1417.019. 18591465 10.1196/annals.1417.019

[16] Mong JA, Pfaff DW. Hormonal and genetic influences underlying arousal as it drives sex and aggression in animal and human brains. Neurobiol Aging. 2003;24:S83–S8. 10.1016/S0197-4580(03)00053-8. 12829115 10.1016/s0197-4580(03)00053-8

[17] Garey J, Goodwillie A, Frohlich J, Morgan M, Gustafsson J-A, Smithies O, et al. Genetic contributions to generalized arousal of brain and behavior. Proceedings Natl Acad Sci. 2003;100:11019–22. 10.1073/pnas.1633773100. 10.1073/pnas.1633773100 PMC196919 12930901

[18] Aston-Jones G, Cohen JD. An integrative theory of locus coeruleus-norepinephrine function: adaptive gain and optimal performance. Annu Rev Neurosci. 2005;28:403–50. 10.1146/annurev.neuro.28.061604.135709. 16022602 10.1146/annurev.neuro.28.061604.135709

[19] Bangasser DA, Wiersielis KR, Khantsis S. Sex differences in the locus coeruleus-norepinephrine system and its regulation by stress. Brain Res. 2016;1641:177–88. 10.1016/j.brainres.2015.11.021. 26607253 10.1016/j.brainres.2015.11.021 PMC4875880

[20] Lubbers LS, Zafian PT, Gautreaux C, Gordon M, Alves SE, Correa L, et al. Estrogen receptor (ER) subtype agonists alter monoamine levels in the female rat brain. J Steroid Biochem Mol Biol. 2010;122:310–7. 10.1016/j.jsbmb.2010.08.005. 20800684 10.1016/j.jsbmb.2010.08.005

[21] Zhang Z, Cordeiro Matos S, Jego S, Adamantidis A, Séguéla P. Norepinephrine drives persistent activity in prefrontal cortex via synergistic α1 and α2 adrenoceptors. PLoS ONE. 2013;8:e66122 10.1371/journal.pone.0066122. 23785477 10.1371/journal.pone.0066122 PMC3681776

[22] Berridge CW, O’Neill J. Differential sensitivity to the wake-promoting actions of norepinephrine within the medial preoptic area and the substantia innominata. Behav Neurosci. 2001;115:165–74. 10.1037/0735-7044.115.1.165. 11256440 10.1037/0735-7044.115.1.165

[23] Morgan MA, Schulkin J, Pfaff DW. Estrogens and non-reproductive behaviors related to activity and fear. Neurosci Biobehav Rev. 2004;28:55–63. 10.1016/j.neubiorev.2003.11.017. 15036933 10.1016/j.neubiorev.2003.11.017

[24] Klump KL. Puberty as a critical risk period for eating disorders: a review of human and animal studies. Horm Behav. 2013;64:399–410. 10.1016/j.yhbeh.2013.02.019. 23998681 10.1016/j.yhbeh.2013.02.019 PMC3761220

[25] Hildebrandt T, Alfano L, Tricamo M, Pfaff DW. Conceptualizing the role of estrogens and serotonin in the development and maintenance of bulimia nervosa. Clin Psychol Rev. 2010;30:655–68. 10.1016/j.cpr.2010.04.011. 20554102 10.1016/j.cpr.2010.04.011 PMC2910148

[26] Ladouceur CD, Kerestes R, Schlund MW, Shirtcliff EA, Lee Y, Dahl RE. Neural systems underlying reward cue processing in early adolescence: the role of puberty and pubertal hormones. Psychoneuroendocrinology. 2019;102:281–91. 10.1016/j.psyneuen.2018.12.016. 30639923 10.1016/j.psyneuen.2018.12.016 PMC7085287

[27] Op de Macks ZA, Bunge SA, Bell ON, Wilbrecht L, Kriegsfeld LJ, Kayser AS, et al. Risky decision-making in adolescent girls: the role of pubertal hormones and reward circuitry. Psychoneuroendocrinology. 2016;74:77–91. 10.1016/j.psyneuen.2016.08.013. 27591399 10.1016/j.psyneuen.2016.08.013

[28] Bullmore E, Sporns O. Complex brain networks: graph theoretical analysis of structural and functional systems. Nat Rev Neurosci. 2009;10:186–98. 10.1038/nrn2575. 19190637 10.1038/nrn2575

[29] Stam CJ, Reijneveld JC. Graph theoretical analysis of complex networks in the brain. Nonlinear Biomed Phys. 2007;1:3. 10.1186/1753-4631-1-3. 17908336 10.1186/1753-4631-1-3 PMC1976403

[30] Hutchison RM, Womelsdorf T, Allen EA, Bandettini PA, Calhoun VD, Corbetta M, et al. Dynamic functional connectivity: promise, issues, and interpretations. Neuroimage. 2013;80:360–78. 10.1016/j.neuroimage.2013.05.079. 23707587 10.1016/j.neuroimage.2013.05.079 PMC3807588

[31] Long Y, Liu X, Liu Z. Temporal stability of the dynamic resting-state functional brain network: current measures, clinical research progress, and future perspectives. Brain Sci. 2023;13:429 10.3390/brainsci13030429. 36979239 10.3390/brainsci13030429 PMC10046056

[32] Sonneville KR, Calzo JP, Horton NJ, Field AE, Crosby RD, Solmi F, et al. Childhood hyperactivity/inattention and eating disturbances predict binge eating in adolescence. Psychol Med. 2015;45:2511–20. 10.1017/S0033291715000148. 26098685 10.1017/S0033291715000148 PMC4655585

[33] Oliva R, Morys F, Horstmann A, Castiello U, Begliomini C. Characterizing impulsivity and resting-state functional connectivity in normal-weight binge eaters. International J Eat Disord. 2020;53:478–88. 10.1002/eat.23212. 10.1002/eat.23212 31868249

[34] Garavan H, Bartsch H, Conway K, Decastro A, Goldstein RZ, Heeringa S, et al. Recruiting the ABCD sample: design considerations and procedures. Developmental Cogn Neurosci. 2018;32:16–22. 10.1016/j.dcn.2018.04.004. 10.1016/j.dcn.2018.04.004 PMC6314286 29703560

[35] Marcus DS, Harms MP, Snyder AZ, Jenkinson M, Wilson JA, Glasser MF, et al. Human connectome project informatics: quality control, database services, and data visualization. Neuroimage. 2013;80:202–19. 10.1016/j.neuroimage.2013.05.077. 23707591 10.1016/j.neuroimage.2013.05.077 PMC3845379

[36] Herting MM, Uban KA, Gonzalez MR, Baker FC, Kan EC, Thompson WK, et al. Correspondence between perceived pubertal development and hormone levels in 9-10 year-olds from the adolescent brain cognitive development study. Front Endocrinol (Lausanne). 2020;11:549928 10.3389/fendo.2020.549928. 33679599 10.3389/fendo.2020.549928 PMC7930488

[37] Kaufman J, Birmaher B, Brent D, Rao UMA, Flynn C, Moreci P, et al. Schedule for affective disorders and schizophrenia for school-age children-present and lifetime version (K-SADS-PL): initial reliability and validity data. Journal Am Acad Child Adolesc Psychiatry. 1997;36:980–8. 10.1097/00004583-199707000-00021. 9204677 10.1097/00004583-199707000-00021

[38] Baranger DAA, Finsaas MC, Goldstein BL, Vize CE, Lynam DR, Olino TM. Tutorial: power analyses for interaction effects in cross-sectional regressions. Advances Methods Pract Psychological Sci. 2023;6:25152459231187531. 10.1177/25152459231187531. 10.1177/25152459231187531 PMC12341451 40799847

[39] Uban KA, Horton MK, Jacobus J, Heyser C, Thompson WK, Tapert SF, et al. Biospecimens and the ABCD study: rationale, methods of collection, measurement and early data. Dev Cogn Neurosci. 2018;32:97–106. 10.1016/j.dcn.2018.03.005. 29606560 10.1016/j.dcn.2018.03.005 PMC6487488

[40] Carskadon MA, Acebo C. A self-administered rating scale for pubertal development. Journal Adolesc Health. 1993;14:190–5. 10.1016/1054-139X(93)90004-9. 10.1016/1054-139x(93)90004-9 8323929

[41] Casey BJ, Cannonier T, Conley MI, Cohen AO, Barch DM, Heitzeg MM, et al. The adolescent brain cognitive development (ABCD) study: imaging acquisition across 21 sites. Developmental Cogn Neurosci. 2018;32:43–54. 10.1016/j.dcn.2018.03.001. 10.1016/j.dcn.2018.03.001 PMC5999559 29567376

[42] Harvey PO, Le Bastard G, Pochon JB, Levy R, Allilaire JF, Dubois B, et al. Executive functions and updating of the contents of working memory in unipolar depression. J Psychiatr Res. 2004;38:567–76. 10.1016/j.jpsychires.2004.03.003. 15458852 10.1016/j.jpsychires.2004.03.003

[43] Nikolin S, Tan YY, Schwaab A, Moffa A, Loo CK, Martin D. An investigation of working memory deficits in depression using the n-back task: A systematic review and meta-analysis. J Affect Disord. 2021;284:1–8. 10.1016/j.jad.2021.01.084. 33581489 10.1016/j.jad.2021.01.084

[44] Chaarani B, Hahn S, Allgaier N, Adise S, Owens MM, Juliano AC, et al. Baseline brain function in the preadolescents of the ABCD Study. Nat Neurosci. 2021;24:1176–86. 10.1038/s41593-021-00867-9. 34099922 10.1038/s41593-021-00867-9 PMC8947197

[45] Fonov V, Evans AC, Botteron K, Almli CR, McKinstry RC, Collins DL. Unbiased average age-appropriate atlases for pediatric studies. Neuroimage. 2011;54:313–27. 10.1016/j.neuroimage.2010.07.033. 20656036 10.1016/j.neuroimage.2010.07.033 PMC2962759

[46] Power JD, Barnes KA, Snyder AZ, Schlaggar BL, Petersen SE. Spurious but systematic correlations in functional connectivity MRI networks arise from subject motion. Neuroimage. 2012;59:2142–54. 10.1016/j.neuroimage.2011.10.018. 22019881 10.1016/j.neuroimage.2011.10.018 PMC3254728

[47] Satterthwaite TD, Wolf DH, Loughead J, Ruparel K, Elliott MA, Hakonarson H, et al. Impact of in-scanner head motion on multiple measures of functional connectivity: relevance for studies of neurodevelopment in youth. Neuroimage. 2012;60:623–32. 10.1016/j.neuroimage.2011.12.063. 22233733 10.1016/j.neuroimage.2011.12.063 PMC3746318

[48] Fan L, Li H, Zhuo J, Zhang Y, Wang J, Chen L, et al. The human brainnetome atlas: a new brain atlas based on connectional architecture. Cereb Cortex. 2016;26:3508–26. 10.1093/cercor/bhw157. 27230218 10.1093/cercor/bhw157 PMC4961028

[49] Zhang Z, Telesford QK, Giusti C, Lim KO, Bassett DS. Choosing wavelet methods, filters, and lengths for functional brain network construction. PLoS ONE. 2016;11:e0157243 10.1371/journal.pone.0157243. 27355202 10.1371/journal.pone.0157243 PMC4927172

[50] Cao M, Halperin JM, Li X. Abnormal functional network topology and its dynamics during sustained attention processing significantly implicate post-TBI attention deficits in children. Brain Sci. 2021;11:1348. 34679412 10.3390/brainsci11101348 PMC8533973

[51] Achard S, Bullmore E. Efficiency and cost of economical brain functional networks. PLoS Comput Biol. 2007;3:e17 10.1371/journal.pcbi.0030017. 17274684 10.1371/journal.pcbi.0030017 PMC1794324

[52] Handwerker DA, Roopchansingh V, Gonzalez-Castillo J, Bandettini PA. Periodic changes in fMRI connectivity. Neuroimage. 2012;63:1712–9. 10.1016/j.neuroimage.2012.06.078. 22796990 10.1016/j.neuroimage.2012.06.078 PMC4180175

[53] Braun U, Schäfer A, Walter H, Erk S, Romanczuk-Seiferth N, Haddad L, et al. Dynamic reconfiguration of frontal brain networks during executive cognition in humans. Proceedings Natl Acad Sci. 2015;112:11678–83. 10.1073/pnas.1422487112. 10.1073/pnas.1422487112 PMC4577153 26324898

[54] Omidvarnia A, Zalesky A, Mansour L S, Van De Ville D, Jackson GD, Pedersen M. Temporal complexity of fMRI is reproducible and correlates with higher order cognition. Neuroimage. 2021;230:117760. 10.1016/j.neuroimage.2021.117760. 33486124 10.1016/j.neuroimage.2021.117760

[55] Cao M, Wu Z, Li X. GAT-FD: An integrated MATLAB toolbox for graph theoretical analysis of task-related functional dynamics. PLoS ONE. 2022;17:e0267456 10.1371/journal.pone.0267456. 35446912 10.1371/journal.pone.0267456 PMC9022818

[56] Alves PN, Forkel SJ, Corbetta M, Thiebaut de Schotten M. The subcortical and neurochemical organization of the ventral and dorsal attention networks. Communications Biol. 2022;5:1343 10.1038/s42003-022-04281-0. 10.1038/s42003-022-04281-0 PMC9729227 36477440

[57] Yeo BT, Krienen FM, Sepulcre J, Sabuncu MR, Lashkari D, Hollinshead M, et al. The organization of the human cerebral cortex estimated by intrinsic functional connectivity. J Neurophysiol. 2011;106:1125–65. 10.1152/jn.00338.2011. 21653723 10.1152/jn.00338.2011 PMC3174820

[58] Poskanzer C, Aly M. Switching between external and internal attention in hippocampal networks. J Neurosci. 2023;43:6538 10.1523/JNEUROSCI.0029-23.2023. 37607818 10.1523/JNEUROSCI.0029-23.2023 PMC10513067

[59] Deshpande G, Zhao X, Robinson J. Functional parcellation of the hippocampus based on its layer-specific connectivity with default mode and dorsal attention networks. Neuroimage. 2022;254:119078. 10.1016/j.neuroimage.2022.119078. 35276366 10.1016/j.neuroimage.2022.119078

[60] Wold S, Ruhe A, Wold H, Dunn IWJ. The collinearity problem in linear regression. the partial least squares (PLS) approach to generalized inverses. SIAM J Sci Stat Comput. 1984;5:735–43. 10.1137/0905052.

[61] Wold S, Sjöström M, Eriksson L. PLS-regression: a basic tool of chemometrics. Chemometrics Intell Lab Syst. 2001;58:109–30. 10.1016/S0169-7439(01)00155-1.

[62] Igelström KM, Graziano MSA. The inferior parietal lobule and temporoparietal junction: A network perspective. Neuropsychologia. 2017;105:70–83. 10.1016/j.neuropsychologia.2017.01.001. 28057458 10.1016/j.neuropsychologia.2017.01.001

[63] Balodis IM, Kober H, Worhunsky PD, White MA, Stevens MC, Pearlson GD, et al. Monetary reward processing in obese individuals with and without binge eating disorder. Biol Psychiatry. 2013;73:877–86. 10.1016/j.biopsych.2013.01.014. 23462319 10.1016/j.biopsych.2013.01.014 PMC3686098

[64] Grahn JA, Parkinson JA, Owen AM. The cognitive functions of the caudate nucleus. Prog Neurobiol. 2008;86:141–55. 10.1016/j.pneurobio.2008.09.004. 18824075 10.1016/j.pneurobio.2008.09.004

[65] Kanoski SE, Grill HJ. Hippocampus contributions to food intake control: mnemonic, neuroanatomical, and endocrine mechanisms. Biol Psychiatry. 2017;81:748–56. 10.1016/j.biopsych.2015.09.011. 26555354 10.1016/j.biopsych.2015.09.011 PMC4809793

[66] Contreras-Rodríguez O, Martín-Pérez C, Vilar-López R, Verdejo-Garcia A. Ventral and dorsal striatum networks in obesity: link to food craving and weight gain. Biol Psychiatry. 2017;81:789–96. 10.1016/j.biopsych.2015.11.020. 26809248 10.1016/j.biopsych.2015.11.020

[67] Bodell LP, Wildes JE, Goldschmidt AB, Lepage R, Keenan KE, Guyer AE, et al. Associations between neural reward processing and binge eating among adolescent girls. J Adolesc Health. 2018;62:107–13. 10.1016/j.jadohealth.2017.08.006. 29054735 10.1016/j.jadohealth.2017.08.006 PMC5742026

[68] Krentzel AA, Willett JA, Johnson AG, Meitzen J. Estrogen receptor alpha, G-protein coupled estrogen receptor 1, and aromatase: developmental, sex, and region-specific differences across the rat caudate-putamen, nucleus accumbens core and shell. J Comp Neurol. 2021;529:786–801. 10.1002/cne.24978. 32632943 10.1002/cne.24978 PMC7775873

[69] Willett JA, Cao J, Johnson A, Patel OH, Dorris DM, Meitzen J. The estrous cycle modulates rat caudate-putamen medium spiny neuron physiology. Eur J Neurosci. 2020;52:2737–55. 10.1111/ejn.14506. 31278786 10.1111/ejn.14506 PMC6943200

[70] Lewitus VJ, Blackwell KT Estradiol receptors inhibit long-term potentiation in the dorsomedial striatum. eNeuro. 2023;10. 10.1523/eneuro.0071-23.2023 10.1523/ENEURO.0071-23.2023 PMC10405883 37487741

[71] Lewitus VJ, Kim J, Blackwell KT. Sex and estradiol effects in the rodent dorsal striatum. Eur J Neurosci. 2024;60:6962–86. 10.1111/ejn.16607. 39573926 10.1111/ejn.16607 PMC11647445

[72] Hoek HW. Incidence, prevalence and mortality of anorexia nervosa and other eating disorders. Curr Opin Psychiatry. 2006;19:389–94. 16721169 10.1097/01.yco.0000228759.95237.78

[73] Holsen LM, Zarcone JR, Thompson TI, Brooks WM, Anderson MF, Ahluwalia JS, et al. Neural mechanisms underlying food motivation in children and adolescents. Neuroimage. 2005;27:669–76. 10.1016/j.neuroimage.2005.04.043. 15993629 10.1016/j.neuroimage.2005.04.043 PMC1535274

