# Supplementary material for: Distinct attention network topology and dynamics and their relations with pubertal hormones in preadolescent boys and girls with binge eating
Source: Transl Psychiatry. 2025 Nov 22;16:5. doi: 10.1038/s41398-025-03756-y (PMC12783676; doi:10.1038/s41398-025-03756-y)
Supplement: Supplementary file 1 — Supplementary Materials [file 41398_2025_3756_MOESM1_ESM.docx]

Supplementary Materials

**Emotional N-Back Task**

The emotional N-back is an adaptation of the original N-back design in which participants view a series of stimuli and must remember if the stimuli match that which they were presented two trials ago (2-back trials), or that which they were presented at the beginning of the block (0-back trials). 0-back trials are included to model sustained attention without an added working memory component. In the current study we used 0-back trials only. These trials were completed as follows. Upcoming 0-back blocks were indicated via an instruction screen before the block started. Each block contained 10 trials, each of which lasted for 2.5 seconds. In each block, 2 targets were presented (e.g. the matching stimulus to the stimulus shown at the beginning of the block). To answer correctly, participants correctly identified the when the current stimulus matched that shown at the start of the screen. In 75% of the blocks, the stimulus was a human face showing either a neutral, negative or positive emotion. In the remaining trials, the stimulus used was a location. Responses were made on a button pad with two buttons: one to indicate a match and one to indicate a non-match.^1^

**Binary Network Threshold Determination**

To determine the appropriate cost threshold range for the functional network, network global and local efficiency were calculated over a cost range from 0.1 to 0.5 with the increment of 0.01. Network global efficiency reflects network integration of distributed nodes, defined as:

$$E_{global}\left( G \right)= \frac{1}{n\left( n-1 \right)} \sum_{ij\in Gj\neq i} \frac{1}{d_{ij}}$$

Where *n* was the number of nodes, and *d_ij_* was the inverse of the shortest path length between nodes *i* and *j*.

Network local efficiency reflects network segregation, defined as:

$$E_{local}\left( G \right)= \frac{1}{n}\sum_{i\in G} E_{global}\left( G_{i} \right)$$

Where G_i_ was a subnetwork consisting of all neighboring nodes of node *i,* and global efficiency was calculated using the equation above.

Functional brain networks are characterized by a small-world architecture, which provides high global and local efficiency of parallel information processing while maintaining low network cost^2,3^. A brain network is considered to be a small-world network if it meets the criteria: *Eglobal(Gregular)* < *Eglobal(G)* < *Eglobal(Grandom*) and *Elocal(Grandom)* < *Elocal(G)* < *Elocal(Gregular),* where *Eglobal(Gregular)*, *Eglobal(Grandom)*, *Elocal(Gregular)* and *Elocal(Grandom)* represent the global and local efficiency of node- and edge-matched regular and random networks, respectively.

**Definition of efficiency, degree and betweenness centrality**

Nodal efficiency was defined as:

$$E_{nodal}(i)=\frac{1}{n-1}\sum_{j\in N j\neq i} \frac{1}{d_{ij}}$$

Where *n* was the number of nodes, and *d_ij_* was the inverse of the shortest path length between nodes *i* and *j*.

Betweenness-centrality was defined as:

$$B\left( i \right)=\frac{1}{\left( n-1 \right)\left( n-2 \right)}\sum_{j,k \in N j\neq k} \frac{p\left( i | j,k \right)}{P\left( j,k \right)}$$

Where *j, k* were pairs of nodes, *p(i|j,k)* was the probability that the shortest path between *j* and *k* passes through *i*, and *P(j,k)* was the total number of shortest paths between *j* and *k.*

Nodal degree is simply the number of connections (edges) that a node has. For each participant, the values of nodal efficiency, betweenness-centrality and degree of each node were averaged over the network cost range.

Figure S1. Summarized interaction effect power calculations from 1000 simulations.


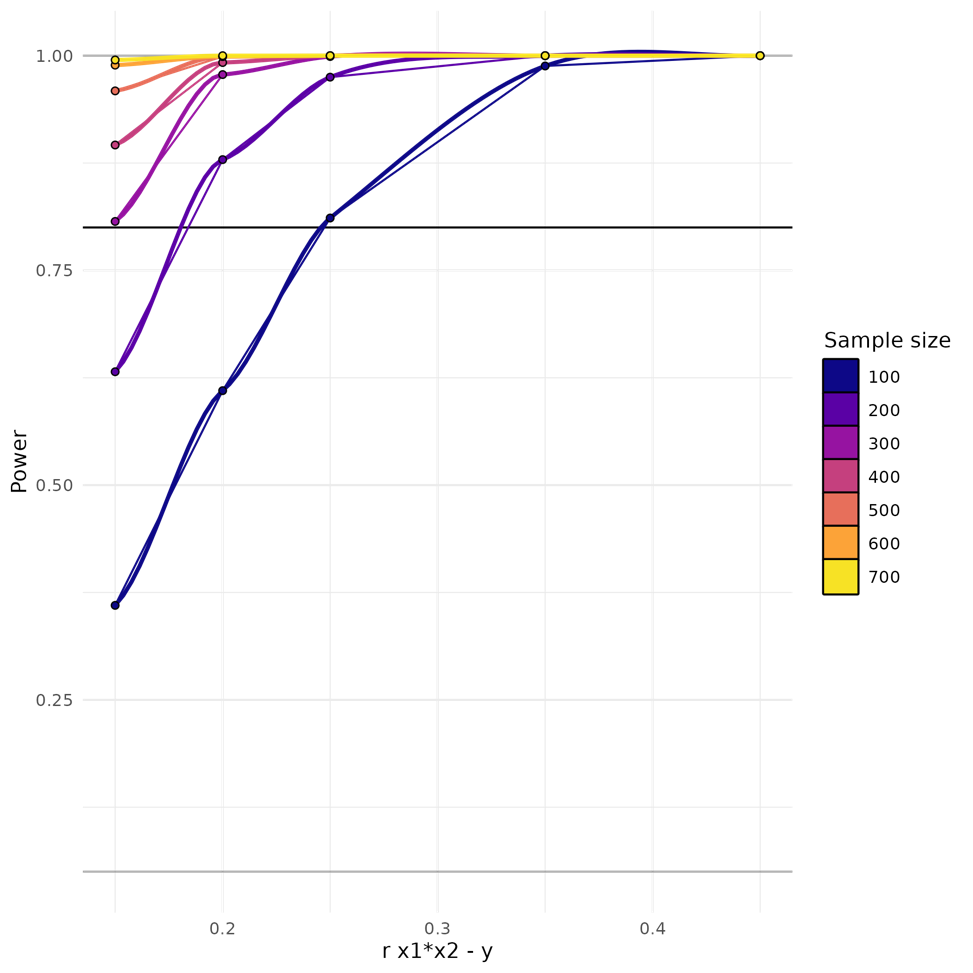


Figure S2. Summarized main group effect power calculations


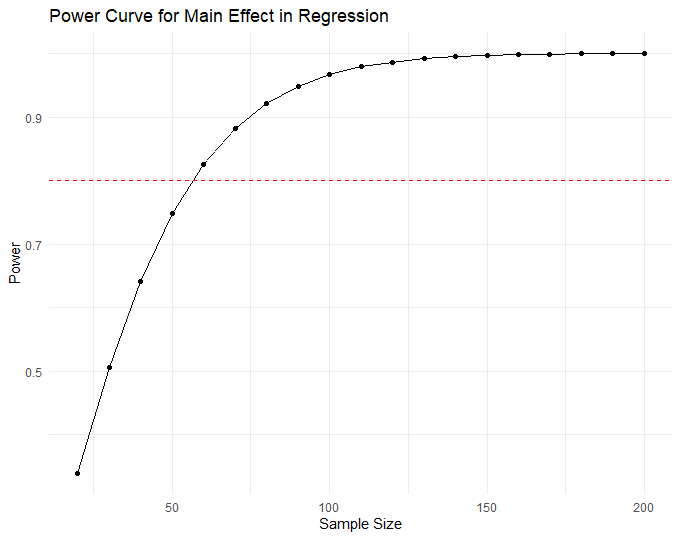


Table S1. Group comparison of static nodal topological properties prior to Bonferroni correction.

|  | **Region** | | **BE** **vs CON** | | | **BE**  **M(SD)** | **CON**  **M(SD)** | **p_uncorrected_** | **p_BON_** | **partial eta^2^** |
| --- | --- | --- | --- | --- | --- | --- | --- | --- | --- | --- |
| ***Efficiency*** | |  | |  |  | |  |  |  |  |
|  | | L Precentral Gyrus | | ↑ | .51 (.09) | | .50 (.09) | .038 | - | .0129 |
|  | | L Inferior Parietal Gyrus | | ↑ | .53 (.11) | | .50 (11) | .044 | NS | .0121 |
|  | | R Hippocampus | | ↑ | .46 (.11) | | .42 (.14) | .019 | NS | .0165 |
|  | | **L Hippocampus** | | **↑** | **.48 (.11)** | | **.43 (.12)** | **.002** | **.03** | **.0276** |
|  | | R Globus Pallidus | | ↑ | .56 (.08) | | .53 (.09) | .0002 | - | .0393 |
|  | | L Putamen | | ↑ | .552 (.08) | | .546 (.09) | .046 | NS | .0118 |
|  | | **L Caudate** | | **↑** | **.57 (.08)** | | **.55 (.10)** | **<.001** | **<.001** | **.0494** |
|  | | R Occipital Thalamus | | ↑ | .49 (.11) | | .47 (.12) | .013 | NS | .0185 |
| ***Degree*** | |  | |  |  | |  |  |  |  |
|  | | L Orbitofrontal Gyrus | | ↓ | 18.6 (4.9) | | 20.3 (4.5) | .009 | - | .0205 |
|  | | **L Hippocampus** | | **↑** | **10.8 (5.5)** | | **8.6 (4.7)** | **.002** | **.03** | **.0277** |
|  | | R Globus Pallidus | | ↑ | 15.3 (5.8) | | 13.9 (5.4) | .002 | - | .0275 |
|  | | **L Caudate** | | **↑** | **16.4 (4.9)** | | **15.1 (5.9)** | **<.001** | **.01** | **.0338** |
|  | | R Occipital Thalamus | | ↑ | 11.3 (5.6) | | 10.1 (5.8) | .030 | NS | .0147 |
|  | | L Inferior Frontal Gyrus | | ↓ | 15.9 (5.6) | | 16.3 (5.9) | .045 | NS | .0121 |
|  | | L Precentral Gyrus | | ↓ | 16.7 (4.8) | | 18.4 (5.5) | .02 | NS | .0162 |
| ***BC*** | |  | |  |  | |  |  |  |  |
|  | | L Middle Frontal Gyrus | | ↑ | 85.1 (53.0) | | 70.3 (52.2) | .044 | - | .012 |
|  | | R Inferior Frontal Gyrus | | ↓ | 67.0 (48.0) | | 77.0 (57.8) | .027 | NS | .015 |
|  | | **L Inferior Parietal Gyrus** | | **↑** | **64.0 (58.6)** | | **41.8 (38.7)** | **.001** | **.015** | **.031** |
|  | | R Hippocampus | | ↑ | 32.7 (38.5) | | 25.5 (30.2) | .007 | NS | .021 |

Abbreviations: L, left; R, right; BE, Participants with Binge Eating; CON, control; P_BON_, p-values Bonferroni corrected for network, Symptoms; BC, betweenness-centrality. Significant results following Bonferroni correction are shown in bold.

Table S2. Group comparison of Variance of nodal topological properties during different substages of sustained attention processing.

|  | **Region** | **BE**  **vs CON** | **BE**  **M(SD)** | **CON**  **M(SD)** | **p_uncorrected_** | **p_BON_** | **partial eta^2^** |
| --- | --- | --- | --- | --- | --- | --- | --- |
| ***Substage of Attention Initiation*** | | | | | | |  |
| **Efficiency** | R Inferior Parietal Gyrus | ↓ | .007 (.005) | .008 (.006) | .016 | NS | .017 |
|  | L Inferior Parietal Gyrus | ↓ | .007 (.005) | .008 (.006) | .019 | NS | .016 |
| **Degree** | **R Inferior Parietal Gyrus** | **↓** | **37.7 (27.8)** | **45.0 (30.1)** | **.003** | **.045** | **.026** |
|  | R Superior Frontal Gyrus | ↓ | 37.2 (23.4) | 43.2 (30.2) | .033 | NS | .013 |
| **BC** | R Middle Frontal Gyrus | ↑ | 3774.0 (5818) | 2895 (3250) | .012 | NS | .019 |
|  | R Occipital Gyrus | ↓ | 2790.8 (2609) | 3435.5 (3668.1) | .016 | - | .017 |
| ***Substage of Stable Attention Processing*** | | | | | | |  |
| **Efficiency** | R Caudate | ↓ | .006 (.003) | .007 (.004) | .006 | NS | .022 |
|  | L Superior Frontal Gyrus | ↑ | .007 (.004) | .006 (.003) | .015 | NS | .017 |
|  | L Caudate | ↓ | .007 (.004) | .008 (.004) | .033 | NS | .013 |
|  | R Thalamus | ↓ | .006 (.004) | .007 (.004) | .048 | NS | .012 |
| **Degree** | R Superior Frontal Gyrus | ↓ | 30.5(14.0) | 34.6(17.0) | .012 | NS | .018 |
|  | L Superior Frontal Gyrus | ↑ | 35.9 (18.1) | 32.7(18.5) | .041 | NS | .012 |
| **BC** | **R Middle Frontal Gyrus** | **↑** | **3750.7 (3000.3)** | **2860.8(1832.7)** | **.003** | **.045** | **.027** |
|  | R Insula | ↓ | 3438.8 (2118.4) | 4090.5 (3942.9) | .011 | NS | .019 |
|  | R Caudate | ↑ | 4126.8 (3374.2) | 3477.8(258.0) | .033 | NS | .013 |
|  | R Thalamus | ↓ | 3695.9(3145.9) | 4375.8(4282.5) | .049 | NS | .011 |
| ***Substage of Post-Attention*** | | | | | | |  |
| **Efficiency** | L Middle Frontal Gyrus | ↓ | .006 (.004) | .007 (.004) | .006 | NS | .022 |
|  | R Orbitofrontal Cortex | ↓ | .006 (.004) | .007 (.005) | .010 | NS | .020 |
|  | L Hippocampus | ↓ | .007 (.004) | .008 (.005) | .010 | NS | .020 |
|  | R Hippocampus | ↓ | .007(.004) | .008(.005) | .033 | NS | .013 |
|  | R Amygdala | ↓ | .007(.004) | .008 (.005) | .045 | NS | .012 |
| **Degree** | R Superior Frontal Gyrus | ↓ | 34.5 (16.3) | 40.5 (21.8) | .007 | NS | .021 |
|  | R Orbitofrontal Cortex | ↓ | 32.9 (21.5) | 39.2 (22.4) | .008 | NS | .020 |
|  | L Middle Frontal Gyrus | ↓ | 31.7 (18.8) | 35.89(20.5) | .017 | NS | .017 |
|  | R Amygdala | ↓ | 32.6 (20.0) | 37.4 (22.8) | .037 | NS | .013 |
|  | L Inferior Frontal Gyrus | ↓ | 34.6 (17.5) | 39.3 (20.3) | .047 | NS | .012 |
| **BC** | L Inferior Parietal Gyrus | ↑ | 4171 (4702) | 3224 (2684) | .022 | NS | .015 |
|  | L Middle Frontal Gyrus | ↑ | 4194 (5089) | 3303(3351) | .044 | NS | .012 |
| ***Substage of Resting State*** | | | | | | |  |
| **Degree** | L Inferior Parietal Gyrus | ↓ | 34.2 (21.0) | 38.9 (23.2) | .044 | NS | .012 |
| **BC** | **L Inferior Parietal Gyrus** | **↑** | **4169 (5763)** | **2894 (2530)** | **.003** | **.045** | **.026** |
|  | R Middle Frontal Gyrus | ↑ | 3308 (3382) | 2516 (2129) | .007 | NS | .022 |

Abbreviations: L, left; R, right; BE, Participants with Binge Eating; CON, control; P_BON_, p-values Bonferroni corrected for network; NS, non-significant; BC, betweenness-centrality. Significant results following Bonferroni correction are shown in bold.

Table S3. Group-by-sex post-hoc comparisons of Variance of nodal topological properties during different substages of sustained attention processing.

|  | **Region** | **Interaction**  **p_uncorrected_** | **Interaction**  **p_BON_** | **Post-hoc Boys BE** **vs CON** | **Post-hoc Girls BE** **vs CON** |
| --- | --- | --- | --- | --- | --- |
| ***Substage of Attention Initiation*** | | | | | |
| **Efficiency** | L Inferior Frontal Gyrus | .014 | NS |  |  |
|  | **R Precentral Gyrus** | **.001** | **.015** | ↓ | NS |
|  | R Fusiform Gyrus | .013 | NS |  |  |
|  | L Inferior Parietal Gyrus | .036 | NS |  |  |
|  | R Globus Pallidus | .009 | NS |  |  |
| **Degree** | L Inferior Frontal Gyrus | .009 | NS |  |  |
|  | L Orbitofrontal Gyrus | .044 | NS |  |  |
|  | R Precentral Gyrus | .031 | NS |  |  |
| **BC** | R Middle Temporal Gyrus | .034 | NS |  |  |
| ***Substage of Stable Attention Processing*** | | | | | |
| **Efficiency** | R Superior Frontal Gyrus | .022 | NS |  |  |
|  | L Superior Frontal Gyrus | .015 | NS |  |  |
|  | L Precentral Gyrus | .013 | NS |  |  |
|  | R Precentral Gyrus | .023 | NS |  |  |
|  | R Insula | .015 | NS |  |  |
| **Degree** | L Inferior Frontal Gyrus | .046 | NS |  |  |
|  | L Orbitofrontal Gyrus | .044 | NS |  |  |
|  | **R Precentral Gyrus** | **.002** | **.03** | ↓ | NS |
|  | L Inferior Parietal Gyrus | .031 | NS |  |  |
|  | R Insula | .015 | NS |  |  |
| **BC** | R Middle Frontal Gyrus | .026 | NS |  |  |
| ***Substage of Post-Attention*** | | | | | |
| **BC** | L Precentral Gyrus | .049 | NS |  |  |
|  | L Inferior Parietal Gyrus | .021 | NS |  |  |
|  | L Caudate | .01 | NS |  |  |
| ***Substage of Resting State*** | | | | | |
| **Efficiency** | **R Precentral Gyrus** | **.002** | **.03** | ↓ | **↑** |
|  | R Inferior Parietal Gyrus | .004 | NS |  |  |
|  | R Insula | .009 | NS |  |  |
| **Degree** | L Superior Frontal Gyrus | .026 | NS |  |  |
|  | R Precentral Gyrus | .015 | NS |  |  |
| **BC** | **L Hippocampus** | **.002** | **.03** | **↑** | NS |
|  | R Insula | .038 | NS |  |  |

Abbreviations: L, left; R, right; BE, Participants with Binge Eating; CON, control; P_BON_, p-values Bonferroni corrected for network; NS, non-significant; BC, betweenness-centrality. Significant results following Bonferroni correction are shown in bold.1. Casey, B.J.*, et al.* The Adolescent Brain Cognitive Development (ABCD) study: Imaging acquisition across 21 sites. *Developmental Cognitive Neuroscience* **32**, 43-54 (2018).

2. Bullmore, E. & Sporns, O. Complex brain networks: graph theoretical analysis of structural and functional systems. *Nature Reviews Neuroscience* **10**, 186-198 (2009).

3. Achard, S. & Bullmore, E. Efficiency and Cost of Economical Brain Functional Networks. *PLOS Computational Biology* **3**, e17 (2007).
